# Deep learning algorithm predicts diabetic retinopathy progression in individual patients

**DOI:** 10.1038/s41746-019-0172-3

**Published:** 2019-09-20

**Authors:** Filippo Arcadu, Fethallah Benmansour, Andreas Maunz, Jeff Willis, Zdenka Haskova, Marco Prunotto

**Affiliations:** 1 Roche Informatics, Roche, Basel, Switzerland; 2 Roche Personalized Healthcare, Roche, Basel, Switzerland; 3 Clinical Science Ophthalmology, Genentech, Inc., South San Francisco, CA USA; 4 Roche Personalized Healthcare, Genentech, Inc., South San Francisco, CA USA; 5 Immunology, Infectious Disease & Ophthalmology, Roche, Basel, Switzerland; 6 School of Pharmaceutical Sciences, University of Geneva, Geneva, Switzerland

**Keywords:** Vision disorders, Predictive markers, Predictive markers, Macular degeneration

## Abstract

The global burden of diabetic retinopathy (DR) continues to worsen and DR remains a leading cause of vision loss worldwide. Here, we describe an algorithm to predict DR progression by means of deep learning (DL), using as input color fundus photographs (CFPs) acquired at a single visit from a patient with DR. The proposed DL models were designed to predict future DR progression, defined as 2-step worsening on the Early Treatment Diabetic Retinopathy Diabetic Retinopathy Severity Scale, and were trained against DR severity scores assessed after 6, 12, and 24 months from the baseline visit by masked, well-trained, human reading center graders. The performance of one of these models (prediction at month 12) resulted in an area under the curve equal to 0.79. Interestingly, our results highlight the importance of the predictive signal located in the peripheral retinal fields, not routinely collected for DR assessments, and the importance of microvascular abnormalities. Our findings show the feasibility of predicting future DR progression by leveraging CFPs of a patient acquired at a single visit. Upon further development on larger and more diverse datasets, such an algorithm could enable early diagnosis and referral to a retina specialist for more frequent monitoring and even consideration of early intervention. Moreover, it could also improve patient recruitment for clinical trials targeting DR.

## Introduction

**Fig. 1:**
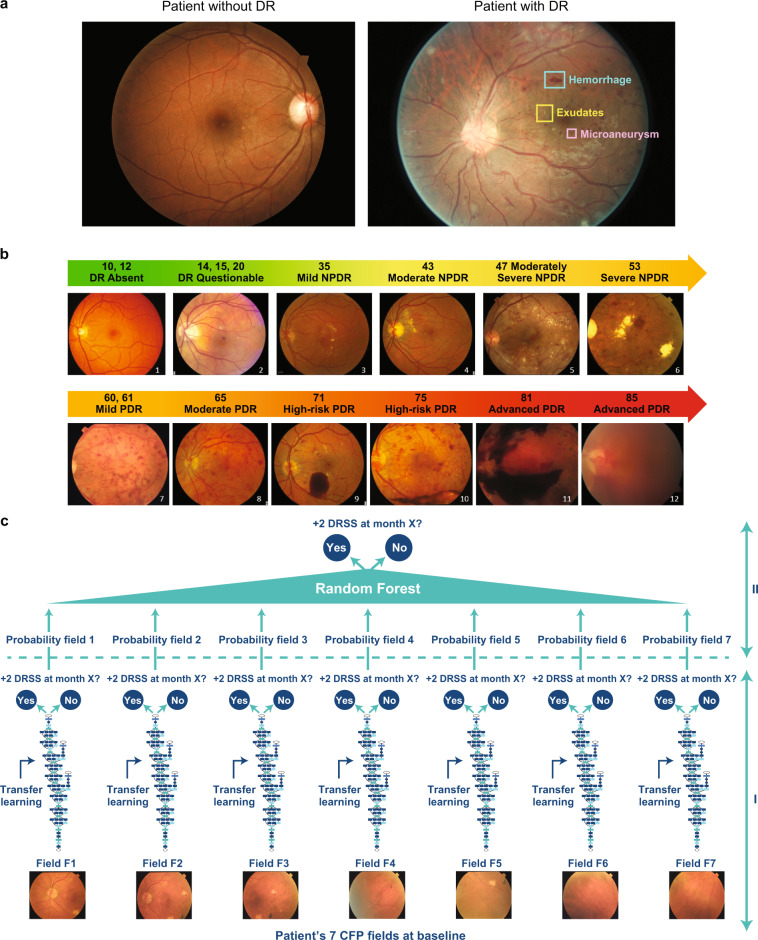
An overview of retinal imaging features analyzed to assess diabetic retinopathy (DR) severity and a schematic of the study design. **a** Example of fovea-centered color fundus photographs (CFPs) of a patient without DR (left) and a patient with signs of DR (right). In the CFP of the patient with signs of DR (right), one example each of hemorrhage, exudate, and a microaneurysm are highlighted. Both examples have been selected from the Kaggle DR dataset.^47^ **b** Schematic of the Diabetic Retinopathy Severity Scale (DRSS) established by the Early Treatment Diabetic Retinopathy Study (ETDRS) group to measure DR worsening over time. **c** Schematic of the two-phase modeling to detect two-step or more DRSS worsening over time. In phase I, field-specific Inception-v3 deep convolutional neural networks (DCNNs) called “field-specific DCNNs” or “pillars” are trained by means of transfer learning to predict whether the patient will progress two ETDRS DRSS steps. In phase II, the probabilities independently generated by the field-specific DCNNs are aggregated by means of random forest

Vision loss due to diabetic eye disease is on the rise and it is expected to reach epidemic proportions globally in the next few decades. In 2017, ~425 million people worldwide had diabetes, and this number is estimated to increase to 642 million by 2040.^1^ Diabetic retinopathy (DR) is the most common and insidious microvascular complication of diabetes, and can progress asymptomatically until a sudden loss of vision occurs. Almost all patients with type 1 diabetes mellitus and ~60% of patients with type 2 diabetes mellitus will develop retinopathy during the first 20 years from onset of diabetes.^2^ However, DR often remains undetected until it progresses to an advanced vision-threatening stage. The current state of DR screening in the real world, based on assessment of color fundus photographs (CFPs, see Fig.1a) by a retina specialist or a trained grader, leaves a large proportion of patients undiagnosed and therefore receiving medical help too late, in part due to low adherence and access to retina screening visits.^3,4^ In-person expert examinations are impractical and unsustainable given the pandemic size of the diabetic population.^5–7^ Notwithstanding, early detection and prevention of DR progression are essential to mitigate the rising threat of DR.

Artificial intelligence (AI) may offer a solution to this conundrum. Deep learning (DL), and specifically, deep convolutional neural networks (DCNNs),^8^ can be used for an end-to-end assessment of raw medical images to produce a target outcome prediction. The diagnostic use of DCCN algorithms is already spreading in various healthcare areas,^9,10^ such as radiology,^11,12^ dermatology,^13^ and pathology.^14^ In ophthalmology, groundbreaking work has recently been conducted on the automation of DR grading^15–17^ and prediction of cardiovascular risk factors^18^ by DCNN analysis of CFPs.

The purpose of this work was to go beyond the use of DL for DR diagnostics^15–17,19^ and to assess the feasibility of developing DCNNs operating on 7-field CFPs that can predict the future threat of significant DR worsening at a patient level over a span of 2 years after the baseline visit.

To achieve that, our DCNNs have been trained on high-quality 7-field CFPs acquired at a single visit and graded for DR severity by masked and well-trained reading center experts, using the validated Early Treatment Diabetic Retinopathy Study (ETDRS) Diabetic Retinopathy Severity Scale (DRSS)^20^ from large controlled clinical trials. Previous studies have limited the deployment of DCNNs to fovea- or optic nerve–centered CFPs.^15–19^ Our findings highlight the importance of the predictive signal located in the peripheral retinal fields of patients with DR and suggest that such a predictive algorithm, upon further development and proper validation, could help fight blindness by identifying fast DR progressors for referral to a retina specialist or inclusion in clinical trials targeting early stages of DR.

## Results

### Model performance

**Fig. 2:**
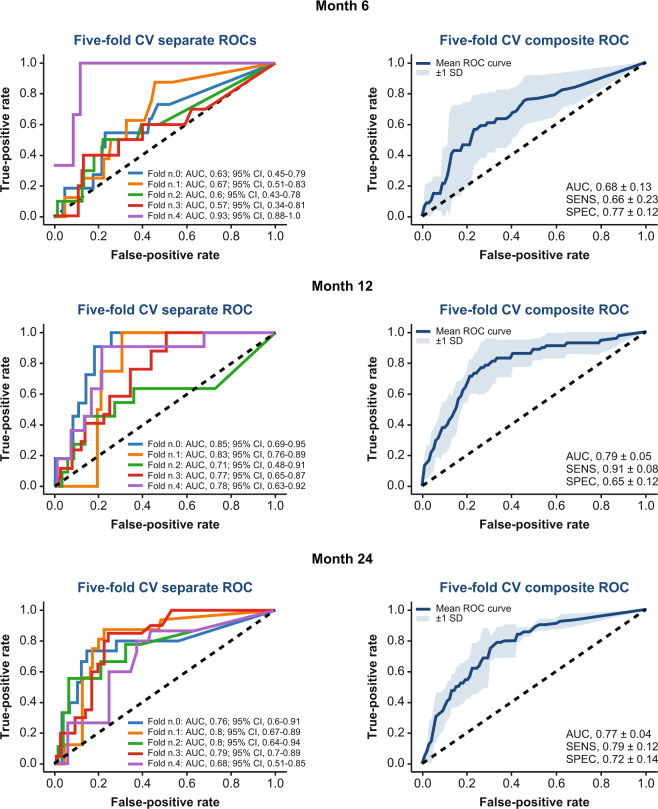
Summary of the results for the prediction of two-step or more diabetic retinopathy progression at months 6, 12, and 24 using 7-field color fundus photographs of patients at baseline. AUC area under the curve, CI confidence interval, CV cross-validation, ROC receiver operating characteristic, SD standard deviation, SENS sensitivity, SPEC specificity

The DL models (the architecture is shown in Fig. 1c and described in detail in the Methods section) were able to predict 2-step or more ETDRS DRSS worsening at 6, 12, and 24 months with an area under the curve (AUC) of 0.68 ± 0.13 (sensitivity, 66% ± 23%; specificity, 77% ± 12%), 0.79 ± 0.05 (sensitivity, 91% ± 8%; specificity, 65% ± 12%), and 0.77 ± 0.04 (sensitivity, 79% ± 12%; specificity, 72% ± 14%), respectively. The receiver operating characteristic curves of the fivefold cross-validation (CV) are shown in Fig. 2. By comparing these values with the average AUC of the individual field-specific DCNN models (Table 1), it appears that the aggregation did significantly improve the overall performance, especially for month 12 *(P* = 0.00049) and 24 (*P* = 0.00042). Results for month 6 were weaker compared with months 12 and 24, mainly due to the scarcity of patients with DR progression within the first 6 months (~6%; see details in the “Methods” section).

**Table 1:** Performance of the individual field-specific DCNNs in terms of AUC

Month	F1	F2	F3	F4	F5	F6	F7
6	0.65 ± 0.12	0.65 ± 0.11	0.63 ± 0.09	0.59 ± 0.08	0.72 ± 0.11	0.66 ± 0.14	0.69 ± 0.12
12	0.68 ± 0.04	0.62 ± 0.07	0.67 ± 0.05	0.75 ± 0.06	0.70 ± 0.04	0.72 ± 0.05	0.74 ± 0.03
24	0.69 ± 0.07	0.61 ± 0.06	0.67 ± 0.04	0.68 ± 0.05	0.70 ± 0.03	0.65 ± 0.05	0.74 ± 0.04

The associated errors are the standard deviation over the AUC values of 25 DCNNs (five repetitions × five folds, *n* = 25) trained for each field

*AUC* area under the curve, *DCNN* deep convolutional neural network

Models using just the ETDRS DRSS grade at baseline achieved an AUC ~ 0.52; only slightly above the “tossing-coin line” (Supplementary Fig. 2).

### Predictive value of the individual CFP fields

The optic-nerve-centered field (F1) and the fovea-centered field (F2) are generally regarded as the most important fields in a standard ophthalmoscopy exam. The purpose of this analysis was to evaluate the predictive signal of DR progression in these central retinal fields compared with the peripheral fields (F3, F4, F5, F6, and F7). We found that the main predictive contribution came from the peripheral retinal fields (F3, F4, F5, F6, and F7) that encompass areas of the retina far from both the fovea and optic nerve. Performance comparisons between models trained only on central fields (F1 and F2) versus models trained on all seven retinal fields support our finding. For this comparison, we performed the random forest (RF) aggregation only with the probabilities generated by the F1- and F2-specific DCNNs (Fig. 1c). Using this subset of the RF inputs brought the AUC down to 0.62 ± 0.13 (from 0.68 ± 0.13 with all seven fields; *P* = 0.0486), 0.64 ± 0.05 (from 0.79 ± 0.05 with all seven fields; *P* = 0.00014), and 0.69 ± 0.05 (from 0.77 ± 0.04 with all seven fields; *P* = 0.0023) for months 6, 12, and 24, respectively.

**Fig. 3:**
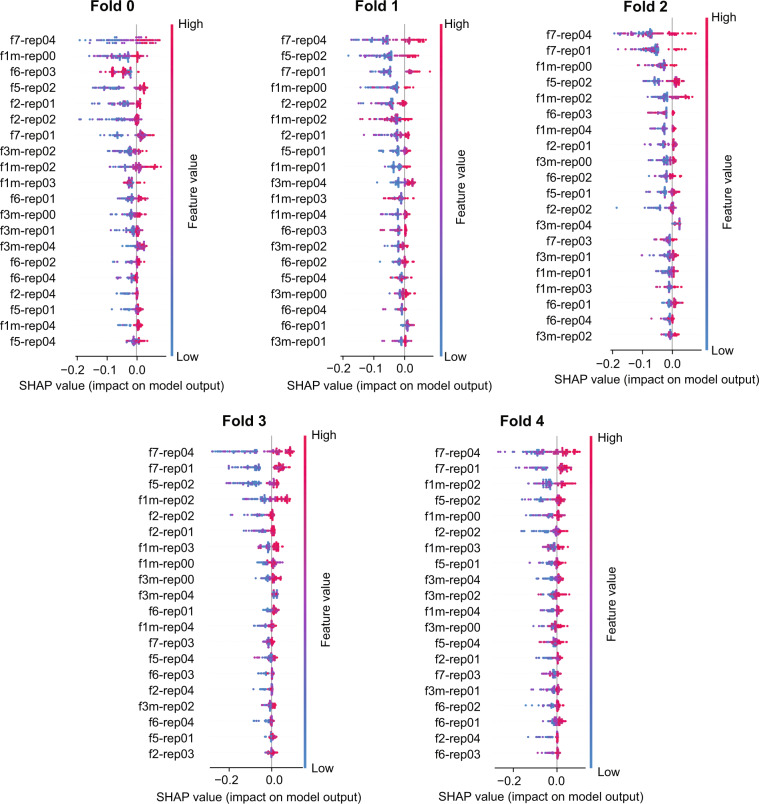
SHAP plots summarizing the pointwise and average contribution of each deep convolutional neural network (DCNN) to the random forest aggregation. SHAP plots outlining the pointwise contribution of each DCNN. In this example, the SHAP analysis is related to the five folds used for the prediction of DR progression at month 24 is showed. The DCNNs are ordered in importance from top to bottom. The naming convention of the DCNNs highlights the field (‘f1,’ ‘f2,’ etc.) and repetition (‘rep00,’ ‘rep01,’ etc.)

The analysis by “Shapley Additive Explanations” (SHAP)^21^ allows for interpretation of predictions of complex symbolic machine learning (ML) models by attributing the descriptors to the weights of importance. Here it was used to evaluate the field-specific DCNN contribution to the final RF aggregation. Figure 3 shows SHAP values on the prediction level for the five folds used for CV, indicating the contribution of the RF input features (namely the probabilities of DR progression generated by all individual DCNNs) for the final prediction. Figure 3 generally confirms that high probabilities (dots in the red spectrum) for individual DCNNs contribute to the prediction of faster DR progression (appear on the positive part of the *x*-axis) and that low probabilities (dots in the blue spectrum) result in the opposite. This pattern is broken in the few cases where the aggregation used low- and high-probability values to predict both classes. This further confirms that using just one field would not be sufficient to properly address the prediction. In this particular case, Fig. 3 highlights that F5- and F7-specific DCNNs play a more crucial role in the prediction compared with F1 and F2.

### Attribution maps

Attribution maps^22–25^ are a powerful strategy to shed light on the complex mechanism inside a single DCNN. These maps highlight areas where the model focuses its attention in order to decide how to classify a certain query image. Attribution maps are useful to check whether the decision mechanism of a DCNN is relatable to human expectation. The maps analyzed in this study were generated by guided back propagation,^24^ which provided the most salient results among different attribution techniques that were tested.

**Fig. 4:**
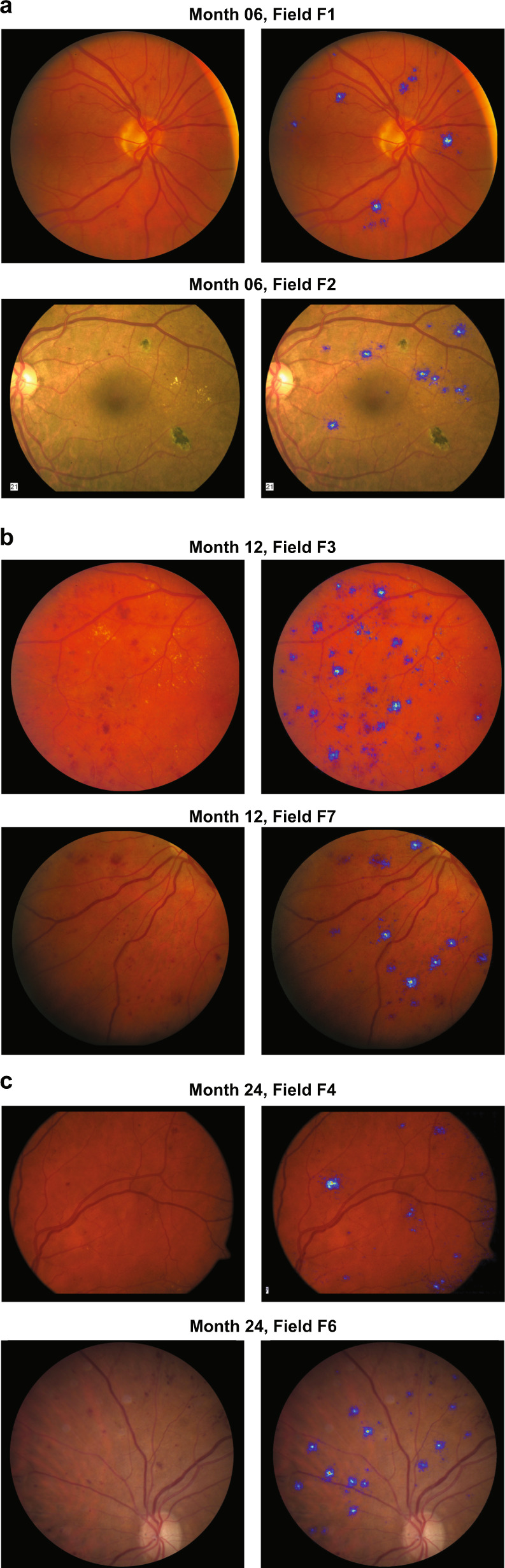
Example of attribution maps placed side by side to the original test color fundus image. In each set, the original image is on the left and the attribution map is on the right. The attribution of the deep convolutional neural networks focuses mainly on microaneurysms, hemorrhages, and exudates. **a** Two examples of attribution maps for the model predicting diabetic retinopathy (DR) progression at month 6. **b** Two examples of attribution maps for the artificial intelligence model predicting DR progression at month 12. **c** Two examples of attribution maps for the model predicting DR progression at month 24

Figure 4 offers examples of different target time points and CFP fields, where test images are placed side-by-side to the corresponding attribution map. In general, the DCNNs seem to focus mainly on hemorrhages and microaneurysms, and partially on exudates, which are well recognized for their association with DR.^26^ A remarkable aspect is that the DCNNs are able to highlight very small and low-contrast microaneurysms, even though they were not explicitly designed to accomplish this task. The correlation between microaneurysms and DR progression has been studied by Piri et al.^27^ and our attribution maps seem to be in line with these studies. However, there is an important difference; in our work, the DCNNs learn a functional mapping from baseline to month X (6, 12, or 24) using a single time point rather than the multiple visits used by Piri et al.^27^ to measure microaneurysm turnover. Please see the Supplement for examples of how attribution maps on the same image vary from month 6 through 24 (Supplementary Fig. 3) or with different repetitions of the prediction model (Supplementary Fig. 4).

Examples highlighted in Fig. 4 indicate that pathologies such as microaneurysms, hemorrhages, and hard exudates could be predictive for DR progression. Future studies may confirm this hypothesis by, for example, replacing DL with symbolic ML methods operating on hand-crafted features based on these pathologies.

## Discussion

This work demonstrates the feasibility of developing a DL algorithm to identify patients who will experience DR worsening by two or more ETDRS DRSS steps over the next 2 years, based solely on CFPs acquired at a single visit. Currently, it is only possible to approximate progression risk for groups of patients with similar signs and symptoms on the basis of their assessed DR severity level, but it is not possible to accurately predict the course of DR in an individual patient.^20^ Previous data from ETDRS studies demonstrated that DR is a slowly progressing disease and that there is an increasing risk of DR worsening and vision-threatening events as DR severity increases.^20^ For example, using the traditional manual grader assessment, a patient with moderately severe non-proliferative DR (NPDR) will have an ~20% risk, whereas a patient with severe NPDR will have an ~50% risk of progressing to proliferative DR (PDR) within 1 year.^20^ However, it is not know which individual patients belong to the subsets of fast versus slow progressors. This type of individual prediction can be achieved by means of the DL algorithms described in this paper.

A second crucial finding of this study was that any imaging-based diagnostic/predictive AI tool for DR should contemplate the inspection of both central and peripheral retina, instead of being limited to the use of CFPs centered around the fovea or optic nerve (F2, in the traditional ETDRS 7-field photography).^15–19^ Moreover, lateral fields may contain predictive signs of DR worsening before the pathology has affected the macula, allowing for prompt referral and, eventually, timely treatment before vision loss occurs. Coupling algorithms such as the one described here with more recent imaging technologies, such as ultrawide field (UWF) photography, might enable the identification of fast DR progressors even earlier in their disease course. The non-mydriatic UWF CFPs^28–30^ were recently validated for DR severity assessments and have the advantage of capturing a view of 200 degrees of the retina with less time and effort compared with the standard 7-field CFP, while providing good to excellent agreement in determining DR severity.^29,30^ Part of our algorithm validation and expansion strategy is to guarantee that the described algorithm will be capable of operating on UWF CFPs as well.

An inherent limitation of the present work is that our DCNNs have been developed and evaluated on two large, identically designed, sham-controlled, double-masked, phase 3 clinical trials (RIDE [NCT00473382] and RISE [NCT00473330]).^31^ The advantage of using clinical trial data is the availability of high-quality standardized imaging formats and processes as well as assessments by masked experts at a centralized reading center. However, this means that, at this time, our work is only applicable to clinical trial populations within a similar range of pre-specified eligibility criteria. Therefore, validation with datasets acquired in the real world will be essential to ensure that these results are reproducible and applicable to the broader DR population; the authors are already tackling this issue. The great advantage of the dataset at our disposal is that it contains a representation of patients across all levels of DR severity (see the distribution of RIDE/RISE patients with respect to the ETDRS DRSS overlaid with the number of 2-step DR progressors in Supplementary Fig. 1). This was key to testing the algorithm reliability across a diverse DR population. To summarize, the following aspects represent crucial limitations of the presented work: (a) relatively small patient population (~530 patients); (b) patient population fulfilling the eligibility criteria of the RIDE and RISE clinical trials, thus not representing the real world population of patients with diabetes; (c) lack of an external validation set; and (d) limited interpretability of the overall prediction models due to the use of DCNN pillars to separately process CFP fields of view. Another potential limitation of the current analyses was the definition of DR progression. The definition we used is based on the clinically relevant magnitude of step-change on the ETDRS DRSS, which is not commonly used in the clinic. Although this scale was designed based on the correlation with increasing risk of vision loss and has been validated and accepted by regulators around the world, our next step is to explore the feasibility of an algorithm that predicts vision loss directly.

Our work represents a step forward in the application of AI to the field of ophthalmology. The DL modeling presented here differs from previously mentioned studies which either required multiple visits, did not employ ML, or focused on current DR severity diagnosis instead of prediction of the future. Additionally, as highlighted before, peripheral CFP fields have not been used in most of the previous ML and DL approaches dedicated to DR diagnostic development.^15–19^

Our findings suggest that deployment of a predictive DR progression algorithm would enable early identification of patients at highest risk of vision loss, allowing timely referral to retina specialists and potential initiation of treatment before irreversible vision loss occurs. In the context of the global diabetes epidemic, new screening tools could have a substantial socioeconomic impact on the healthcare system. The federal savings are projected to reach $624.0 million annually in the United States alone, if we can deploy tools that enable recommended eye screening and ophthalmic care for all diabetes patients.^32^ However, these estimates are based on the ability to expand diagnosis across members of the diabetes population currently lacking access to medical care. To our knowledge, nobody has yet performed an evaluation of cost savings based on predictive screening, which would identify individuals who require immediate follow up and possible early intervention due to impeding near-future progression. Deployment of a predictive algorithm represents therefore an important conceptual leap for the efficient triage of patients at high risk of vision-threatening DR complications and a step towards a personalized approach to medicine, what it also envisaged as “precision medicine.”

Moreover, the identification of fast DR-progressing patients through a predictive DR progression algorithm would have the potential to support the development of new treatments targeting patients with mild and moderate NPDR. Clinical trials based on the traditional endpoint of DR improvement/worsening are considered prohibitively expensive due to size and/or duration. By enriching the clinical trial population with fast DR-progressing individuals, such an AI-based recruitment strategy would increase the chances of success for clinical trials of novel drugs designed to address the unmet need of those members of the early DR population at the greatest risk of progression and vision loss. This is particularly important considering the rising global prevalence of DR and its potential impact on healthcare systems and society.

## Methods

### Dataset

This study is based on the post hoc retrospective analysis of stereoscopic 7-field CFPs obtained from eyes with DR in the RIDE (NCT00473382)^33–35^ and RISE (NCT00473330)^33–35^ phase 3 studies at baseline (start of the studies) that were not treated with anti-vascular endothelial growth factor (VEGF) therapy. RIDE and RISE were registered on 13/05/2007 with the title “A Study of Ranibizumab Injection in Subjects With Clinically Significant Macular Edema (ME) With Center Involvement Secondary to Diabetes Mellitus” and can be accessed via the ICTRP portal at the following URLs: RIDE at http://apps.who.int/trialsearch/Trial2.aspx?TrialID=NCT00473382, RISE at http://apps.who.int/trialsearch/Trial2.aspx?TrialID=NCT00473330.

The objective of this analyses was to generate algorithms that can predict worsening in untreated eyes from baseline over a period of 2 years. From RIDE and RISE, only the baseline images from those eyes that were randomized to sham/untreated groups and the untreated fellow eyes were used for this work. These eyes had the natural course of DR worsening outcomes without anti-VEGF treatment collected at months 6, 12, and 24. There was a total of 529 (683 eyes, 4781 CFPs), 528 (682 eyes, 4774 CFPs), and 499 (645 eyes, 4515 CFPs) patients with untreated eyes who had all seven fields captured on CFP at months 6, 12, and 24. RIDE and RISE were two parallel, identically designed, phase 3, randomized, double-masked clinical trials of ranibizumab in patients with DR with diabetic macular edema. The studies were sham injection controlled for 2 years and followed for an additional year in which all sham patients crossed over to ranibizumab therapy. The study design, eligibility criteria, and core efficacy and safety outcomes of these trials have been previously described.^32–34^ Baseline ETDRS DRSS DR severity in RIDE/RISE sham-treated study and fellow eyes ranged from 10 (absent) to 71 (high-risk PDR) .^20^ The manually detected rates of two-step or more worsening in sham study eyes and fellow eyes at 2 years from baseline were 9.6% and 11.7%, respectively.^33^ The majority of the CFP images were of high quality due to the training requirement for all study site photographers who participated in CFP acquisition. Additionally, the image assessment for the manual severity grading was of the highest attainable quality because it was performed by two masked readers at an accredited fundus photograph reading center supervised by a senior reader in charge of adjudication when needed.

Each patient data point at each visit consists of seven CFP fields that span a 30-degree view of retina. The different fields are indicated with the following codes: F1 (optic nerve centered); F2 (fovea centered); and F3, F4, F5, F6, and F7 (all peripheral fields); all codes correspond to a general ETDRS standard adopted by reading centers.^35^

The trials adhered to the tenets of the Declaration of Helsinki and were Health Insurance Portability and Accountability Act compliant, and the protocols were approved by the study sites’ institutional review boards and ethics committees (or as applicable). Patients provided written informed consent.

### Outcome variable for DR progression

The 7-field CFPs acquired for each patient at baseline were used to train DL models designed to predict, on an individual patient level, two-step or more worsening with respect to the ETDRS DRSS over 2 years, specifically after 6, 12, and 24 months. The problem under study is a binary classification, where ‘0’ means no occurrence of two-step or more ETDRS DRSS worsening at month X (6, 12, or 24), and ‘1’ means the opposite. The incidence of sham study and fellow eyes diagnosed with worsening by two or more ETDRS DRSS steps was ~6% at month 6, ~10% at month 12, and ~12% at month 24. The histograms in Supplementary Fig. 1 show the distribution of the population of sham study and fellow eyes at baseline with respect to the ETDRS DRSS overlaid with the number of DR progressors and non-progressors for month 6, 12, and 24.

The ETDRS DRSS scale has been validated and widely used for objective quantification of retinopathy severity in the clinical trial setting. The landmark trials, Diabetic Retinopathy Study and ETDRS, established that worsening of DR (that is measured by anatomic features on the ETDRS DRSS) is significantly associated with development of severe vision loss.^36^ Moreover, the incidence of clinically significant diabetic macular edema was shown to correlate with the progression of DR on the ETDRS DRSS from NPDR to PDR.^20^ Even just one-step or more DRSS worsening was shown to be associated with a five- to sixfold increased risk of PDR development, and a three- to fourfold risk of developing clinically significant macular edema with a high likelihood of vision loss over the period of 4 years.^37^ However, considering the intergrader variability associated with this scale, two steps or more on the ETDRS DRSS was deemed a more robust outcome variable to use for predictive modeling.

### Modeling

The overall model is a binary classification whose input data points are sets of seven CFP images acquired for a selected eye at baseline. Modeling was performed in two steps: (1) DCNNs were separately trained for each type of CFP field to form the “pillars”; and (2) the probabilities provided by the individual pillars are then aggregated by means of RFs. Single pillars and RFs are trained against the binary outcome variable defined in the previous section. A simple schematic of the model is provided by Fig. 1c.

The Inception-v3 architecture^38^ was used to build the field-specific pillars. A transfer learning^39^ cascade strategy was adopted to create the starting weights; first, the architecture was initialized with Imagenet^40^ weights and trained on the Kaggle DR^41^ dataset to differentiate between CFPs with no DR from those with signs of DR; the weights generated by this last training are then used to initialize the training of the pillars for DR progression.

Transfer learning was performed, first by replacing and training for 10 epochs the last dense layers while keeping all the others fixed, and then by fine-tuning for 50 epochs all layers from the end up to the third one. The Adam optimizer was used with learning rates adapted for the various pillars. A small parameter grid search was conducted to find the optimal learning rates for the pillars. A five-times fivefold CV scheme with patient ID constraint (data points of the eyes of the same patient were constrained to belong to the same CV fold; i.e., both either to the training or testing set) was adopted to generate a total of 25 DCNNs for each pillar.

RFs^42^ were then used to aggregate the probabilities of all pillars. A five-fold CV scheme with the same folds of the previous step was performed. This means that for each fold, 35 probabilities (seven fields × five repetitions for each CV fold) were used as input features for the RF. Please see Supplementary Methods for additional information about the RF models used in this study.

The model performance was measured in terms of AUC, sensitivity, and specificity evaluated at the Youden’s point,^43^ which is obtained by maximizing the difference between the true- and false-positive rate.

SHAP is a technique originally adopted in game theory to determine how much each player in a collaborative game has contributed to its success. In the ML context, each SHAP value measures how much each feature contributes to the target prediction, either in a positive or a negative way.^21^ The traditional feature importance algorithm is based on the Gini index highlights, which features are contributing the most to the prediction across the entire population^44^ and is known in literature to be characterized by multiple biases,^45^ preventing this algorithm to be reliable in many scenarios. Differently from the Gini index, SHAP offers a local perspective by informing on the most predictive features at the level of individual sample.^21^ In the plots of Fig. 3, each row corresponds to an input feature and each colored dot is a sample, whose color ranges from blue to red to inform whether the selected input feature has a low or a high value for the selected sample.

The attribution maps for the individual DL models presented in Fig. 4 were generated by means of a gradient-based technique called guided-backpropagation.^46^ The idea behind gradient-based methods is that the relative importance of the input features is measured by computing the gradient of the output decision with respect to those input features.^47^ This gradient, once back-projected onto the input image, provides an idea of where the CNN has focused on in order to classify the image in a certain way. In particular, guided-backpropagation^46^ is characterized by the suppression of flow of gradients where either the input or incoming gradients are negative.

### Reporting summary

Further information on research design is available in the Nature Research Reporting Summary.

## Supplementary information

Supplementary Information

Reporting summary

## Data Availability

Qualified researchers may request access to individual patient level data through the clinical study data request platform (www.clinicalstudydatarequest.com). Further details on Roche’s criteria for eligible studies are available here (https://clinicalstudydatarequest.com/Study-Sponsors/Study-Sponsors-Roche.aspx). For further details on Roche’s Global Policy on the Sharing of Clinical Information and how to request access to related clinical study documents, see here (https://www.roche.com/research_and_development/who_we_are_how_we_work/clinical_trials/our_commitment_to_data_sharing.htm).

## Code availability

The code to create the FOV-specific CNN pillars is based on Keras using Tensorflow as backend and is available at this public Github repository (https://github.com/arcaduf/nature_paper_predicting_dr_progression). The repository contains the YAML configuration files used to select the training hyper-parameters of each FOV-specific CNN pillar and a detailed README file outlining the content of the folders and how to make use of the scripts.
