# Author Correction: Deep learning algorithm predicts diabetic retinopathy progression in individual patients

**DOI:** 10.1038/s41746-020-00365-5

**Published:** 2020-12-08

**Authors:** Filippo Arcadu, Fethallah Benmansour, Andreas Maunz, Jeff Willis, Zdenka Haskova, Marco Prunotto

**Affiliations:** 1 Roche Informatics, Roche, Basel, Switzerland; 2 Roche Personalized Healthcare, Roche, Basel, Switzerland; 3 Clinical Science Ophthalmology, Genentech, Inc., South San Francisco, CA USA; 4 Roche Personalized Healthcare, Genentech, Inc., South San Francisco, CA USA; 5 Immunology, Infectious Disease & Ophthalmology, Roche, Basel, Switzerland; 6 School of Pharmaceutical Sciences, University of Geneva, Geneva, Switzerland

Correction to: *npj Digital Medicine* 10.1038/s41746-019-0172-3, published online 20 September 2019

In the original version of the published Article, there was a methodological issue which affected the modeling procedure and how the results change once the procedure is properly amended and the corresponding computations re-run. The methodological errors are described in detail below. For transparency, the original figures and table have not been updated in the original version. Additionally, the contact details for Marco Prunotto have changed since publication and have been updated accordingly.

## Methodological issue

In the first modeling step, deep learning (DL) convolutional neural network (CNNs), also called in this context “pillars”, are trained for each field of view (FOV) of the color fundus photographs (CFPs). During the training of each individual CNN, a grid search approach with a 5-fold cross-validation (CV) scheme is used to find the optimal tuple of learning rate for the transfer learning and for the fine tuning phase. Given a tuple of learning rates and a split of 4 folds for training and 1 fold for testing, training is stopped at the epoch when the area under the curve (AUC) peaks with respect to the fold left for testing. The weights of each CNN are therefore decided on the basis of the “testing” folds, which, in reality, is playing the role of a parameter tuning fold.

As a consequence, the performance of the pillars, reported in terms of mean AUC and its standard deviation over the 5 folds, is over optimistic due to the CNNs overfitting the “test folds” at each round of the CV scheme. The overfitting cascades into the second modeling step involving random forest that leverage as input the probabilities produced by the trained CNNs.

## Methodological amendment

In this amended re-run of the modeling work, we maintain the grid search approach of looking for the optimal tuple of learning rates (one for transfer learning, the other for fine tuning) and the strategy of saving the weights of a CNN at the epoch where the AUC computed on the tuning set reaches the maximum value.

Supplementary Fig. 1 shows the nested 5-fold CV scheme adopted for this amended re-run of the modeling work. Given a tuple of learning rates, a fold is selected to be the test or hold-out set, i.e., kept unseen during training, at each of the 5 CV iterations. This leaves 4 folds to be used for training and hyper-parameter tuning. At this point, 4 CNNs are created by rotating each time the fold used for hyper-parameter tuning and the triplet used for training. This results in a total of 20 CNNs trained for each tuple of learning rates.

Once the grid search is completed, the 5 CNNs with highest tuning AUC are selected for a given FOV, fold and split of the training/tuning set. Selecting more than 1 CNN allows the creation of a more populated ensemble scheme, where there are multiple DL models expressing an “opinion” on the DR progression of the given CFP. The procedure is applied to each of the 7 CFP FOVs. At this point, the first modeling step is concluded.

In the second modeling step, the trained CNNs are executed on the respective training and tuning sets to generate the input probabilities to train the random forest (RF) model. The training, tuning, and testing folds used to perform the RF model are the same used throughout the entire modeling work. A grid search approach is used to find the optimal RF hyper-parameters. In particular, the grid search looks for the optimal combination of the minimum number of samples required to split an internal node, the number of trees, whether the best splitting criterion is provided by the Gini or the entropy index, the maximum number of features to consider when looking for the best split, and the minimum number of samples required to be at a leaf node. Among all these RF instances, for a given fold and split of the training/tuning sets, the RF model with the highest tuning AUC is selected. Therefore, the final RF aggregation entails 20 distinct RF instances. These RF instances are applied to the corresponding hold-out set and the probability vectors generated for the same hold-out by the 4 different RF instances are averaged together. With this final probability vector, the testing AUC, sensitivity (SENS), and specificity (SPEC) can be computed for the selected fold. The process is then repeated over the 5 folds leading to the mean values and the corresponding standard deviations of the aforementioned metrics that are reported in Supplementary Table 1. Sensitivity and specificity are evaluated at Youden’s point, as specified in the manuscript. The probabilities of the RF models sharing the same hold-out fold are averaged together before computing the corresponding testing metrics.

Supplementary Table 1 summarizes the modeling results and allows to quantify the difference with the results produced by the faulty methodology and reported by the published manuscript. Once the described source of overfitting is removed from the modeling procedure, the AUC drops substantially, by a value of 0.1 approximately. The main consequence is that the overall predictive power that can be harvested for DR progression from baseline CFPs is considerably inferior than what was claimed on the basis of the previous faulty results.

## Secondary analysis

### Comparison between single-FOV and 7-FOV aggregation

Table 1 reports the performance of the individual FOV-specific CNNs (100 CNNs for each FOV, 5 repetitions × 5 folds × 4 splits training/tuning) when applied to the hold-out folds. The final row of Table 1 shows the pooled mean AUC and standard deviation across the 7 FOVs for the months. The performance of the RF aggregation reported by Supplementary Table 1 is not statistically significant when compared to the pooled mean AUC of Table 1 (*p* value = 0.300 for month 6, *p* value = 0.227 for month 12, *p* value = 0.610 for month 24).

The correct version of Table 1 appears below.

**Table 1:** Mean AUC and standard deviation of the individual FOV-specific CNNs computed over the 5 hold-out sets (first 7 rows) and corresponding pooled mean and standard deviation (last row).

	Month 6	Month 12	Month 24
F1	0.512 ± 0.097	0.573 ± 0.110	0.601 ± 0.068
F2	0.486 ± 0.083	0.605 ± 0.118	0.541 ± 0.034
F3	0.473 ± 0.040	0.535 ± 0.084	0.600 ± 0.096
F4	0.514 ± 0.119	0.666 ± 0.095	0.631 ± 0.068
F5	0.621 ± 0.092	0.638 ± 0.082	0.689 ± 0.062
F6	0.562 ± 0.049	0.634 ± 0.078	0.598 ± 0.090
F7	0.500 ± 0.039	0.634 ± 0.070	0.650 ± 0.081
Mean 7-FOV	0.524 ± 0.047	0.612 ± 0.041	0.616 ± 0.043

The incorrect version of Table 1 appears below.

**Table:** 

Month	F1	F2	F3	F4	F5	F6	F7
6	0.65 ± 0.12	0.65 ± 0.11	0.63 ± 0.09	0.59 ± 0.08	0.72 ± 0.11	0.66 ± 0.14	0.69 ± 0.12
12	0.68 ± 0.04	0.62 ± 0.07	0.67 ± 0.05	0.75 ± 0.06	0.70 ± 0.04	0.72 ± 0.05	0.74 ± 0.03
24	0.69 ± 0.07	0.61 ± 0.06	0.67 ± 0.04	0.68 ± 0.05	0.70 ± 0.03	0.65 ± 0.05	0.74 ± 0.04

### Comparison between central and peripheral fields

Supplementary Table 2 reports the performance of the RF aggregation using as input only the central fields F1 and F2. From these results, we can see that the 7-FOV RF aggregation (Supplementary Table 1) improves the model performance compared to leveraging only the central fields, but not in a statistically significantly way (*p* value for month 6 not needed as the AUC < 0.5, *p* value = 0.098 for month 12, *p* value = 0.194 for month 24).

Figure 3 displays the recomputed pointwise SHAP plots for the 7-FOV RF aggregation, further confirming the fact that the peripheral fields play an important role in the overall prediction.

**Fig. 3:**
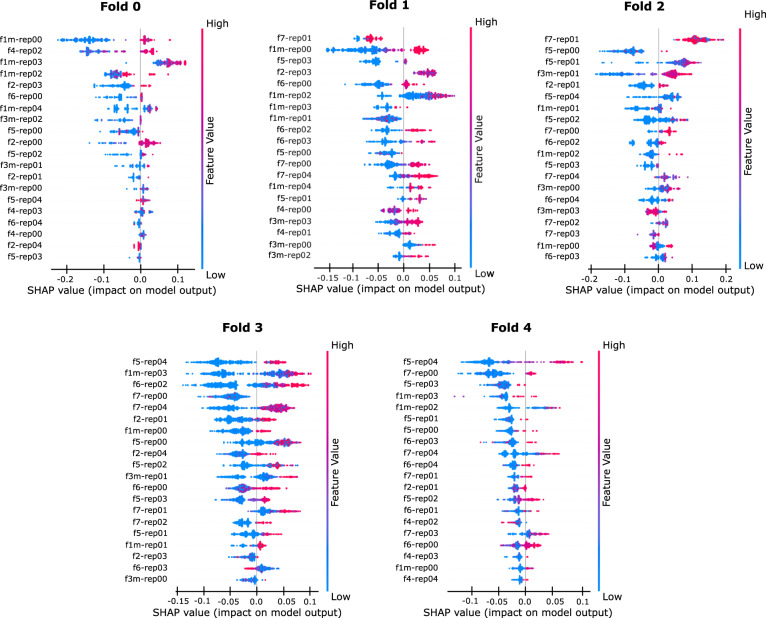
Pointwise SHAP analysis for the 7-FOV RF aggregation of month 12 computed over the hold-out sets.

The correct version of Fig. 3 appears above.

The incorrect version of Fig. 3 appears below.



## Attribution maps

The recomputed attribution maps of samples drawn from the hold-out sets do not present any qualitative difference with respect to those computed with the faulty methodology. Some examples for each month and FOV type are displayed in Fig. 4, as it was done in the original manuscript.

**Fig. 4:**
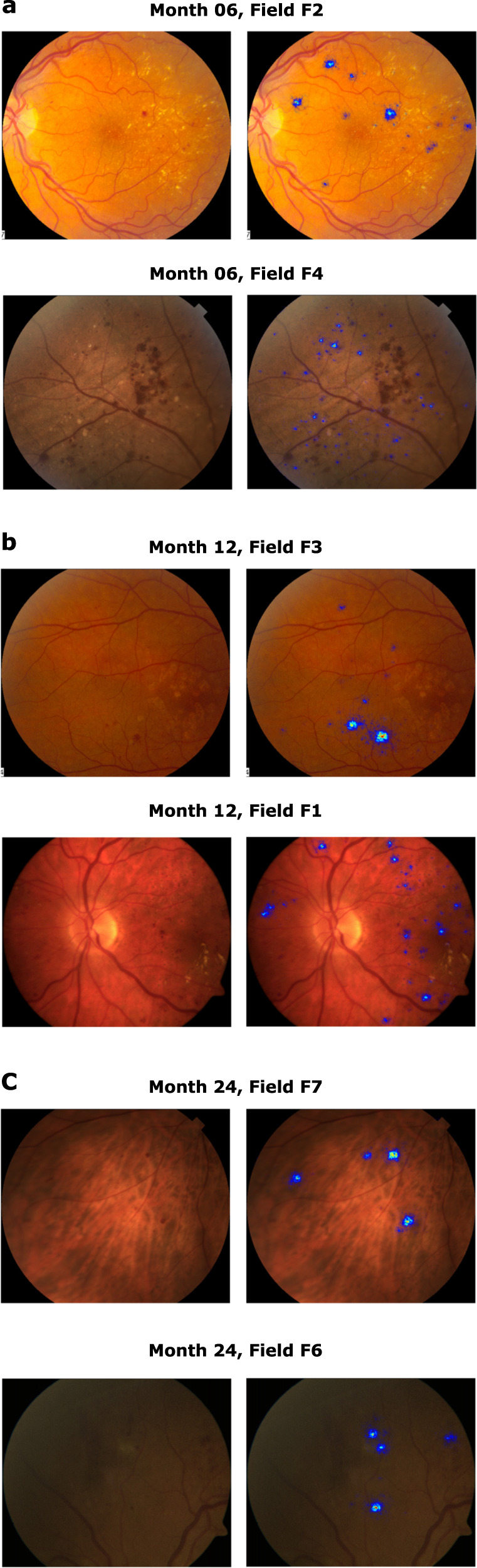
Examples of attribution maps for each month and various types of FOV computed on the hold-out sets.

The correct version of Fig. 4 appears above.

The incorrect version of Fig. 4 appears below.



## Supplementary information

Supplementary Information

