# Supplementary material for: Deep learning algorithm predicts diabetic retinopathy progression in individual patients
Source: NPJ Digit Med. 2019 Sep 20;2:92. doi: 10.1038/s41746-019-0172-3 (PMC6754451; doi:10.1038/s41746-019-0172-3)
Supplement: Supplementary file 1 — Supplementary Information [file 41746_2019_172_MOESM1_ESM.pdf]

Supplementary material for:

Deep learning algorithm for predicting diabetic retinopathy (DR) progression in individual patients

Filippo Arcadu[1,2], Fethallah Benmansour[1,2], Andreas Maunz[1,2], Jeff Willis[3,4], Zdenka Haskova[3,4], and Marco Prunotto[2,5]

[1] Roche Informatics, Roche, Basel, Switzerland.

[2] Roche Personalized Healthcare, Roche, Basel, Switzerland.

[3] Clinical Science Ophthalmology, Genentech, Inc, South San Francisco, CA, United States.

[4] Roche Personalized Healthcare, Genentech, Inc, South San Francisco, CA, United States.

[5] Immunology, Infectious Disease & Ophthalmology, Roche, Basel, Switzerland.

Contents:

Supplementary Figure 1
Supplementary Figure 2
Supplementary Figure 3
Supplementary Figure 4
Supplementary Methods

Supplementary Figure 1

Histograms showing RIDE/RISE study and fellow eyes with (blue) and without (green) a 2-step progression in diabetic retinopathy (DR) severity at months 6 (top), 12 (middle), and 24 (bottom) by baseline DR severity (as measured by the Early Treatment Diabetic Retinopathy Study [ETDRS] Diabetic Retinopathy Severity Scale [DRSS]). In this histogram, DRSS subclasses have been merged together; for example, '35A,' '35B,' '35C,' '35D,' '35E,' and '35F' have been merged into '35.'

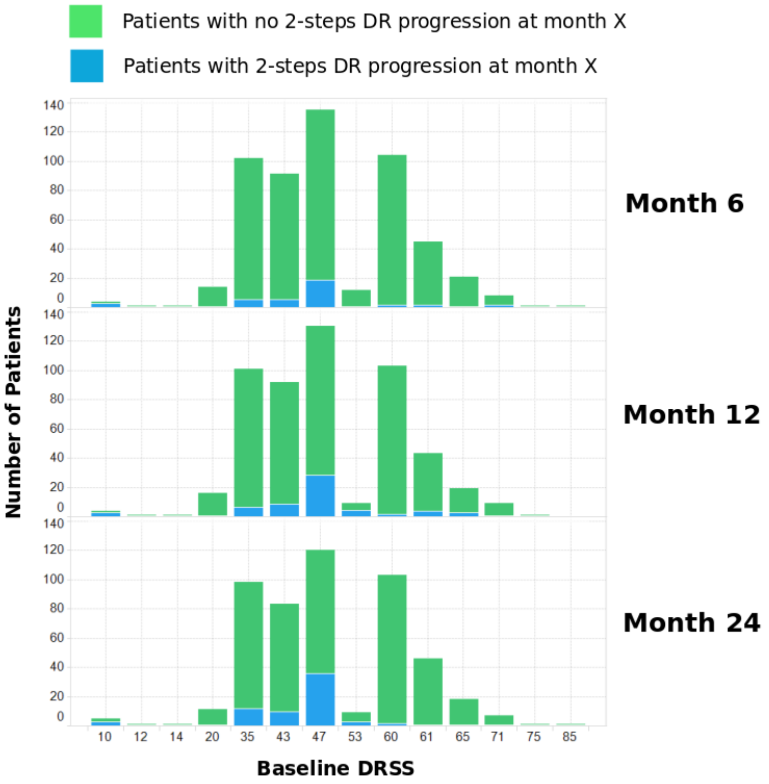

Supplementary Figure 2.

Receiver operating characteristic (ROC) curves of the univariate model to predict 2-step DR progression at months 6 (upper left), 12 (upper right), and 24 (bottom) using only the ETDRS DRSS scores at baseline. OP stands for the Youden's operating point where sensitivity and specificity have been computed. AUC, area under the curve; CI, confidence interval; SENS, sensitivity; SPEC, specificity.

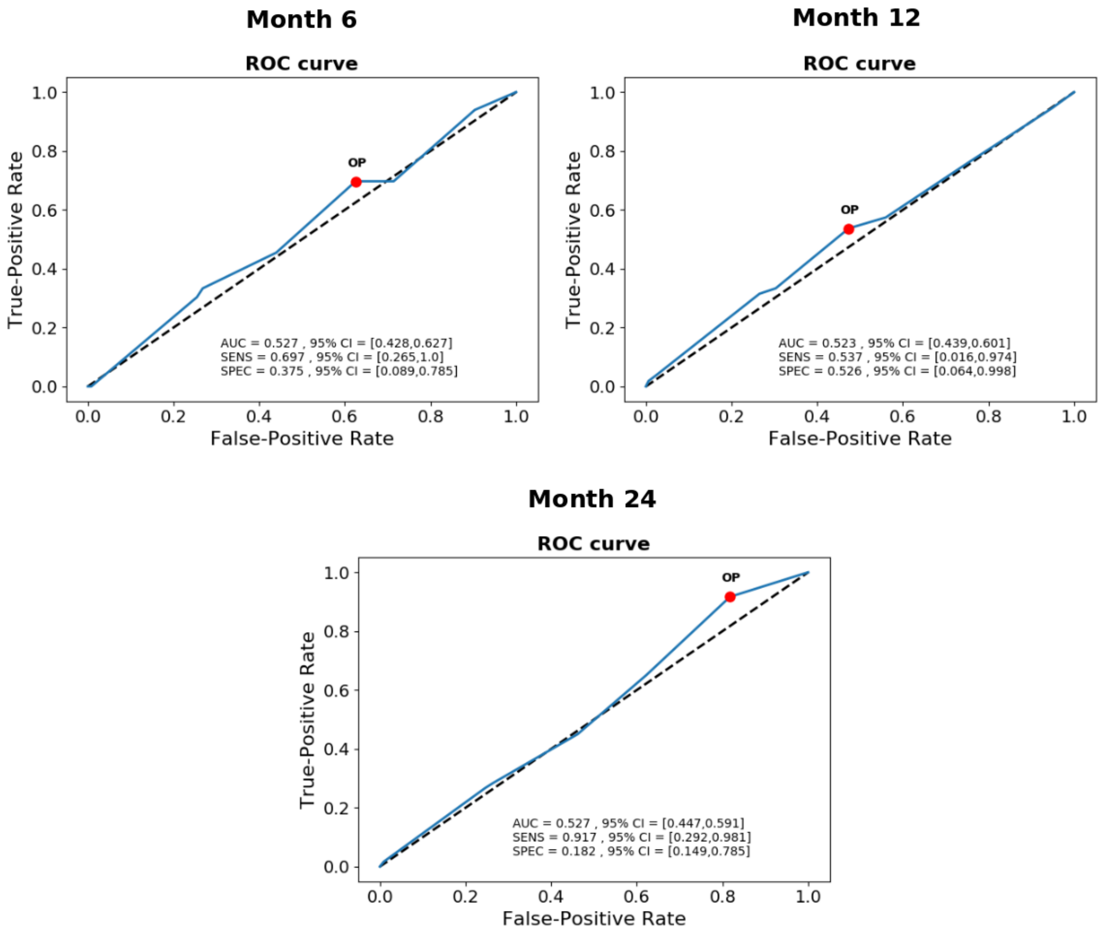

Supplementary Figure 3.

Side-by-side attribution maps related to the same input color fundus photograph and generated by the prediction models at months 6 (left), 12 (middle), and 24 (right). The attribution maps look similar but are not exactly the same. The location of the hotspots seems to be the same but the attention intensity varies, especially from months 6 and 24 to month 12.

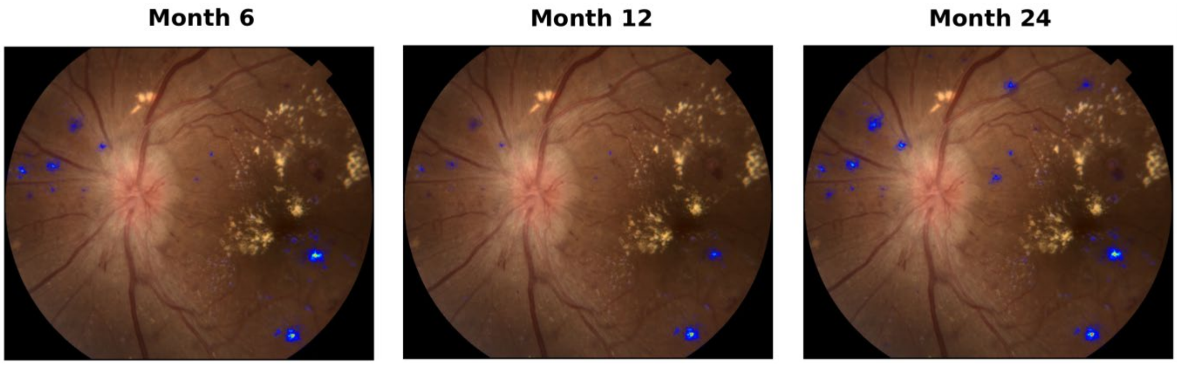

Supplementary Figure 4.

Side-by-side attribution maps related to the same input color fundus photograph and generated by the three different repetitions of the prediction model at month 12. As already seen in Supplementary Figure 3, the attribution maps look relatively similar (in the sense that the hotspot locations are fairly maintained by all maps) but the hotspot intensity may vary substantially. More in-depth studies of this specific matter will determine whether such variations are intrinsic to the guided-backpropagation algorithm or whether there is some aspect peculiar to the specific scientific question addressed in this work.

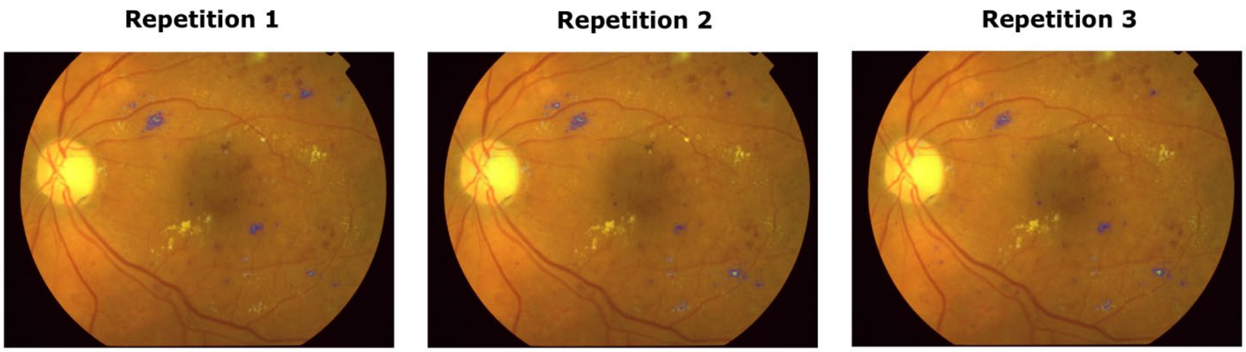

Supplementary Methods

Random forest hyper-parameters

For the random forest (RF), we used the implementation provided by the Python package known as scikit-learn (link). The metric used to choose the best RF model was the AUC. The RF models for all 3 months are expanded until all leaves are pure or until all leaves contain fewer than 2 samples. The RF model at month 6 is characterized by 10 trees, up to 3 features when looking for the best split, a minimum of 5 samples to be a leaf node, and entropy used to measure the splitting quality. The RF model at month 12 is characterized by 15 trees, up to 2 features when looking for the best split, a minimum of 10 samples to be a leaf node, and gini used to measure the splitting quality. The RF model at month 24 is characterized by 10 trees, up to 10 features when looking for the best split, a minimum of 3 features when looking for the best split, and entropy used to measure the splitting quality.
