# Supplementary material for: Author Correction: Deep learning algorithm predicts diabetic retinopathy progression in individual patients
Source: NPJ Digit Med. 2020 Dec 8;3:160. doi: 10.1038/s41746-020-00365-5 (PMC7723990; doi:10.1038/s41746-020-00365-5)
Supplement: Supplementary file 1 — Supplementary Information [file 41746_2020_365_MOESM1_ESM.docx]

Supplementary Information


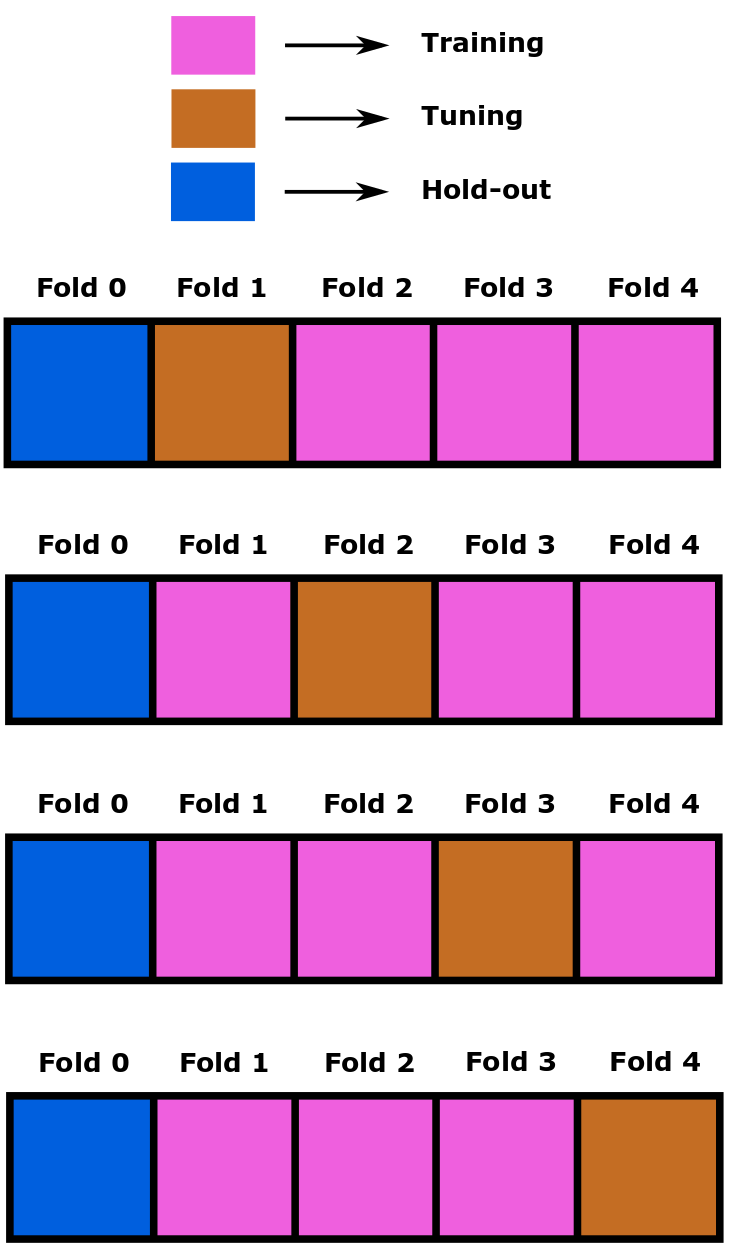


Supplementary Figure 1: Nested 5-fold cross-validation scheme when selecting, e.g., fold n.0 to be the testing or hold-out fold. At this point, 4 CNNs are trained using each time a different tuning folds and a different triplet of training folds. As a result, 4 CNNs are created for each testing fold, leading to an overall amount of 20 models generated by the scheme.

|  | **Month 6** | **Month 12** | **Month 24** |
| --- | --- | --- | --- |
| **Testing AUC** | 0.565 ± 0.068 | 0.658 ± 0.069 | 0.641 ± 0.096 |
| **Testing SENS** | 0.625 ± 0.258 | 0.653 ± 0.131 | 0.701 ± 0.170 |
| **Testing SPEC** | 0.659 ± 0.264 | 0.682 ± 0.116 | 0.625 ± 0.122 |

Supplementary Table 1: AUC, sensitivity and specificity (evaluated at Youden’s point) mean value and standard deviation of the final 7-FOV RF aggregation computed on the testing sets of the nested 5-fold CV scheme.

|  | **Month 6** | **Month 12** | **Month 24** |
| --- | --- | --- | --- |
| **Testing AUC** | 0.483 ± 0.039 | 0.573 ± 0.077 | 0.555 ± 0.096 |

Supplementary Table 2: AUC, sensitivity and specificity (evaluated at Youden’s point) mean value and standard deviation on the final F1-F2 RF aggregation computed on the testing sets of the nested 5-fold CV scheme.
